# Challenges for MicroRNA Microarray Data Analysis

**DOI:** 10.3390/microarrays2020034

**Published:** 2013-03-25

**Authors:** Bin Wang, Yaguang Xi

**Affiliations:** 1 Department of Mathematics and Statistics, University of South Alabama, 411 University BLVD N, Room 325, Mobile, AL 36688, USA; E-Mail: bwang@southalabama.edu; 2 Mitchell Cancer Institute, University of South Alabama, 1660 Springhill Avenue, Mobile, AL 36604, USA

**Keywords:** microRNA, microarray, normalization, measurement error, qRT-PCR, bead array

## Abstract

Microarray is a high throughput discovery tool that has been broadly used for genomic research. Probe-target hybridization is the central concept of this technology to determine the relative abundance of nucleic acid sequences through fluorescence-based detection. In microarray experiments, variations of expression measurements can be attributed to many different sources that influence the stability and reproducibility of microarray platforms. Normalization is an essential step to reduce non-biological errors and to convert raw image data from multiple arrays (channels) to quality data for further analysis. In general, for the traditional microarray analysis, most established normalization methods are based on two assumptions: (1) the total number of target genes is large enough (>10,000); and (2) the expression level of the majority of genes is kept constant. However, microRNA (miRNA) arrays are usually spotted in low density, due to the fact that the total number of miRNAs is less than 2,000 and the majority of miRNAs are weakly or not expressed. As a result, normalization methods based on the above two assumptions are not applicable to miRNA profiling studies. In this review, we discuss a few representative microarray platforms on the market for miRNA profiling and compare the traditional methods with a few novel strategies specific for miRNA microarrays.

## 1. Introduction

miRNAs are a set of naturally-occurring small single-stranded non-coding RNA molecules. They regulate more than 30% of all human genes and mediate gene expression at the post-transcriptional and translational levels in both plants and animals. Many miRNAs are evolutionarily conserved and are involved in many essential biological processes, such as development, cell growth, differentiation, apoptosis and tumorigenesis [1,2,3,4,5,6,7]. The substantial value of miRNAs has been reported for diagnostic and prognostic determination, as well as for eventual therapeutic intervention [8,9]. Although the first miRNA, *lin-4*, was initially discovered over a decade ago, the significance of miRNA research has not been appreciated until recently with the discoveries of thousands of miRNAs in worm, fly and mammalian genomes [10,11,12,13].

Gene expression microarray technology is a very powerful high throughput tool capable of monitoring the expression of thousands of genes in an organism simultaneously [14]. Probe-target hybridization is the central concept to determine the relative abundance of nucleic acid sequences through fluorescence-based detection [15]. In the past few decades, microarrays have been increasingly utilized to investigate the complex molecular interactions in biological systems. Along with increasing interest in miRNAs, most well-established molecular and biological technologies have been successfully transferred into miRNA research, including the microarray and quantitative real-time polymerase chain reaction (qRT-PCR). Currently, there are many commercial miRNA microarray platforms available, including the products from Agilent Technologies, Ambion Inc., Exiqon, Invitrogen, *etc*.

Like most analytical platforms, errors are introduced to miRNA microarrays in almost every single step in the process of acquiring and analyzing microarray data, including sample preparation, sample storage, dying, hybridization, scanning, image processing and equipment errors, among many others. In addition, due to miRNA’s unique signatures, such as small total number and low expression in the majority, the transplantation of traditional normalization methods for mRNA/cDNA profiling analysis is not able to provide a suitable solution for miRNA profiling analysis. The emphasis of this study will be placed on the data analysis techniques applied to the final miRNA expression data profiled with three different platforms: two versions of miRCURY LNA microRNA arrays and one Luminex FlexmiR microRNA Human Panel. The results are validated with the qRT-PCR results by TaqMan Array Human microRNA Panel.

## 2. Measurement Quality and Background Correction

### 2.1. MiRCURY LNA MicroRNA Array

Two versions of miRCURY LNA microRNA arrays are used for miRNA profiling (see the Materials and Methods section for more details). All slides are scanned using an Axon Gene Pix Professional 4200A microarray scanner (Molecular Devices, Sunnyvale, CA, USA), and the images are gridded and analyzed using ImaGene 7.0 software (BioDiscovery Inc., Hawthorne, CA, USA). MiRCURY LNA microRNA Array v7.5.0 (LNAv7 hereafter) is used to profile 359 miRNAs for two HCT-116 cell lines. MiRCURY LNA microRNA Array v9.2 (LNAv9 hereafter) is used to profile 577 miRNAs for 10 osterosarcoma xenograft specimens. On each slide of the LNA arrays, there are four technical replicates for each miRNA. The background signals are estimated by measuring the intensity of the surrounding area (pixels in the local background region) of the corresponding spot masks, and the signal for an miRNA from a specific spot is approximated by the intensity measure from the local signal region. The expression of an miRNA is computed based on the (local) background subtracted signals from the four replicates. All spots are automatically flagged by the image processing software to check the signal quality.

Table 1 shows the five-number summaries of the automatic flags for the spots (probes) for the miRNAs being tested based on thirteen (13) LNAv7 slides and forty-eight (48) LNAv9 slides, respectively. From Table 1, we find the following:

- Among the 13 LNAv7 arrays, on average, approximately 58% of the probes have reasonably strong signals (not flagged). In the worst case, about 42% of spots are not flagged, and approximately 57% of the spots are low-expressed or missing spots. One slide contains more than 77% of spots with no flags.
- Among the 48 LNAv9 arrays, on average, less than 20% of the probes have reasonably strong signals, while more than 50% of the probes are empty spots. The best slides have approximately 45% of non-flagged spots, while the non-flagged spots have less than 4% in the worst slide.
- For both LNAv7 and LNAv9, the proportions of poor spots (background/signal contaminated, high ignored percentage and others) are relatively low.

**Table 1:** Quality flags with miRCURY LNA arrays (all hsa-miR probes).

Version	Type	Min	1st Qu.	Median	Mean	3rd Qu.	Max.
LNAv7	no flag	42.17	50.46	55.68	57.71	62.66	77.42
	empty spots	21.61	36.88	43.99	41.70	49.15	57.51
	poor spots	0.26	0.33	0.46	0.59	0.85	1.17
LNAv9	no flag	3.89	11.18	16.76	18.77	26.80	45.33
	empty spots	52.77	72.03	82.42	80.24	87.98	95.85
	poor spots	0.00	0.61	0.80	1.00	1.38	2.77

In terms of the flagged spots, it looks like LNAv7 arrays have better signal quality than LNAv9 arrays. One potential explanation for the observation is that more weakly or not expressed miRNAs are included in the LNAv9 arrays. To have a closer comparison, we illustrate the summary information for the flags of the 224 miRNAs tested by both LNAv7 and LNAv9 in Table 2. From Table 2, we find that even when comparing the same set of miRNAs among both versions, there was reduced signal in LNAv9 arrays. This difference could also be due to the use of different samples in the experiments: HCT-116 cell lines for LNAv7 and human osterosarcoma xenograft specimens for LNAv9, respectively.

**Table 2:** Quality flags with miRCURY LNA arrays (overlapped hsa-miR probes).

Version	Type	Min.	1st Quartile	Median	Mean	3rd Quartile	Max.
LNAv7	no flag	71.88	96.88	100.00	95.43	100.00	100.00
	empty spots	0.00	0.00	0.00	4.33	3.13	28.125
	poor spots	0.00	0.00	0.00	0.24	0.00	3.13
LNAv9	no flag	9.38	43.75	48.44	48.76	53.13	90.63
	empty spots	3.13	40.63	50.00	46.35	53.13	90.63
	poor spots	0.00	0.00	3.13	4.88	6.25	34.38

### 2.2. FlexmiR MicroRNA Human Panel

A total of 319 human miRNAs are profiled using the bead-based Luminex FlexmiR MicroRNA Human Panel (Luminex, Corp., Austin, TX, USA) for 40 treated and untreated osterosarcoma xenograft specimens. Due to the capacity of the pool, all miRNAs are divided into five groups and are tested using five different human pools separately. The intensities are captured with a Luminex-200 instrument. In addition, for each microsphere type being tested, a background control (water treated in the same manner as an RNA sample) is used to measure the background signal (median fluorescence intensity, MFI). The system does not flag results based on the signal quality. For the 40 profiles based on the treated and untreated samples, we manually flag the signals of different miRNAs as follows: we compute the standard deviation, *s*, of the background signals for each pool. If the signal of an miRNA after background subtraction is smaller than 2s. but not smaller than *s*, we flag the miRNA as weakly expressed; if the signal of an miRNA after background subtraction is smaller than *s*, we flag it as empty; otherwise, an miRNA is not flagged. Table 3 shows the percentages of miRNAs based on all 40 profiles. From Table 3, we find that about ∼60% or more miRNAs have reasonably strong intensity measures across all 40 arrays. In the worst case, the total percentage of weakly or not expressed miRNAs is about 40%. In summary, compared with the LNAv7 and LNAv9 arrays, the signal quality of the bead arrays is much improved, as expected. But on the other hand, we see that the percentage of weakly or not expressed miRNAs is high in arrays from all three platforms.

**Table 3:** Quality flags with FlexmiR bead arrays.

Version	Type	Min.	1st Quartile	Median	Mean	3rd Quartile	Max.
FlexmiR	no flag	59.89	73.70	77.88	77.69	83.65	89.29
	[s,2s)	3.30	5.77	6.73	7.24	8.65	13.46
	< *s*	6.59	10.99	14.97	15.08	18.00	28.85

### 2.3. Signal-to-Noise Ratio

**Figure 1:**
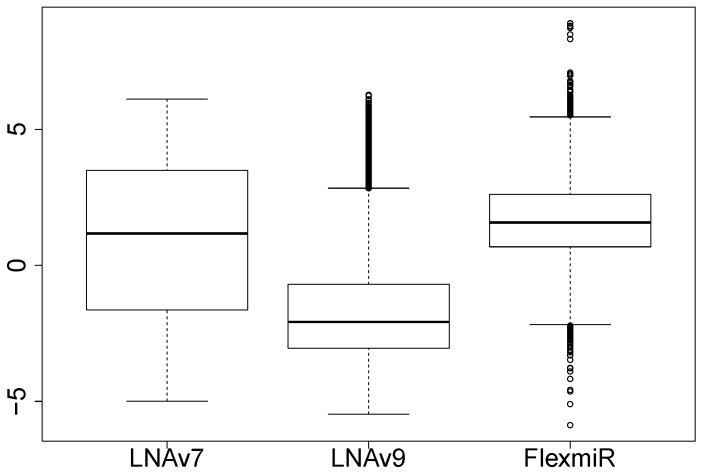
Signal-to-noise ratio comparisons.

We also compute the signal-to-noise ratios (SNRs) based on the signals that are not flagged to compare the signal quality of the three platforms. The ratio of the background-subtracted signal to the local estimated background signal is used to approximate the SNR. Figure 1 shows the boxplots of the logarithms of the SNRs for the LNA arrays and the bead arrays. The two panels to the left in Figure 1 show the boxplots of the probe-level SNRs for human miRNAs from LNAv7 and LNAv9, respectively. The right panel shows the miRNA-level SNRs for all miRNAs from the bead arrays. We find that in terms of SNR, the signal quality of LNAv7 arrays and FlexmiR bead arrays are relatively better than that of LNAv9 arrays. The signal quality of the bead arrays is expected to be better. But the difference between LNAv7 and LNAv9 could mainly be contributed to the different specimens used and some other experimental factors as well.

### 2.4. Background Correction

Let $x=\{x_{1},x_{2},\ldots ,x_{n}\}$ and $y=\{y_{1},y_{2},\ldots ,y_{n}\}$ be the true expressions of the entire miRNA population being tested in two cell populations—a *control sample*, which is an untreated specimen or a specimen treated with saline, and a *test sample*, which is a specimen treated with a drug. Sometimes, the control sample can also be a specimen from a subject without disease, and the test sample is a specimen from a subject with a certain disease. In practice, both *x* and *y* are not observable. Instead, *x* and *y* are usually measured using microarrays or other analytical platforms, and the corresponding measurements. *X* and *Y*. are usually coupled with errors. It is common in practice that *x* and *y* are approximated by simply subtracting the local estimates of the background noises from the intensity measures from the corresponding spots. Or, if replicates exist, the median or mean values will be used for different miRNAs, respectively. For miRNA microarrays, background correction via local background subtraction may cause difficulties in data analysis. First, the majority of miRNAs are weakly or not expressed, and as a result, background-subtraction using local estimates may result in negative values in *x* and *y*, which is not acceptable. Second, ignoring the probes that are flagged as weakly expressed may result in too many missing values or over-estimate the true expression levels of some miRNAs—either way will introduce significant bias to the detection of differentially expressed miRNAs.

It is commonly observed that the standard deviation of the measurements in microarray rises proportionally to the expression level. However, for totally unexpressed genes, this proportionality won’t continue down to zero, due to the fact that measurement errors always exist [16]. Motivated by these observations, two measurement error models are introduced [17,18,19]. Both models describe the measurement error for a given transcript for gene expression arrays using two error components—the multiplicative and additive errors. A generalized logarithm transformation (GLOG) is proposed for gene-expression microarray data analyses [20]. The GLOG can stabilize the variance of the measurements and take care of the measures of the weakly expressed miRNAs quite well and, hence, are suitable for miRNA microarray data analysis (more details in the Materials and Methods section).

## 3. Intra- and Inter-Platform Reproducibility

The diversity of platforms and analytical methods have made cross-platform microarray data comparison and integration challenging. Agreement in results obtained with different platforms have been observed in some comparison studies [21,22,23,24,25,26], while others have not [27,28,29,30]. Various studies tackled the issue of reproducibility of cDNA microarrays across platforms and between laboratories [31,32,33,34,35]. Results showed that the correlation between the two datasets from the Affymetrix platform and the spotted cDNA platform is poor when the ratios and the spot intensities are compared [27,35,36]. The cross-platform reproducibility can be much improved by using standardized protocols [34]. Further, across-laboratory comparisons showed that the reproducibility for a platform within a single laboratory is good, and the data from the best-performing labs agree with each other rather well [31,32]. Using probe sequences matched at the exon level, the consistency of measurements across the different platforms can be improved compared with annotation-based matches [33]. This confirmed the conjecture that the discrepancies might be due to the different array types measuring different variants of the same gene.

Mature miRNAs are short, many of which are approximately 20 nuclides in length. As a result, the inconsistency of measurements caused by annotation-based matches across different miRNA microarray platforms won’t be as severe as those across different mRNA/cDNA microarray platforms. However, the short lengths of the miRNAs will cause a wide Tm range within the entire miRNA population, which results in binding efficacy or fluorescent distortion. This will ultimately influence the sensitivity of different platforms and, hence, make the cross-platform data comparison and data integration a challenge. Kuo *et al.* compared nearly all available commercial and “in-house” platforms and found that the concordance of measurements was lower across platforms than between laboratories on the same platform. In addition, the consistency of low expression miRNAs was lower than that of high expression miRNAs [31,32,33,34,35].

We investigated the intra-platform reproducibility by comparing the Spearman’s correlation coefficients among various miRNA profiles tested on different samples using the same platforms for LNAv9, FlexmiR bead array and TLDA, respectively. Results showed that the bead array has the highest intra-platform reproducibility with a median coefficient of 0.8544 and a standard deviation 0.0475. TLDA and LNAv9 also have reasonable high intra-platform reproducibility, with median coefficients of 0.8118 and 0.7367, respectively. The inter-platform reproducibility between TLDA and the bead array is low, with a median of 0.1060 and a standard deviation of 0.0391. While LNAv9 has relatively higher inter-platform reproducibility with the TLDA (with a median of 0.4872 and a standard deviation of 0.0962) and with bead array (with a median of 0.4521 and a standard deviation of 0.0537) [37]. Overall, the inter-platform reproducibility is much lower compared with the intra-platform reproducibility.

## 4. Normalization

Normalization is an essential step in microarray gene-expression data analysis. It helps to reduce non-biological errors and to convert raw data to valid results. The fundamental assumption of most established normalization methods for high density arrays is that relatively few genes will be dramatically up- or down-regulated compared to the total number of genes, and the intensity measures for the same miRNA population being tested have similar distributions across different slides. However, this assumption is violated for miRNA microarray data, because of the small total number of miRNAs, and the current miRNA microarray platforms possibly do not include enough miRNAs with stable expressions [38].

### 4.1. Linear Normalization

The data obtained from two slides or differently-dyed samples from the same slide might not be directly comparable. For gene expression arrays, the actual expression level in molecular units can hardly be discerned, and hence, it is hard to calibrate data from different arrays. One common normalization method is linear rescaling. That is, a constant is multiplied to all measures from the same array, so that the expression levels of various arrays can be brought roughly to the same levels. The immediate challenge for this approach is how to find the normalizing constants for different arrays. For high density arrays, if the expression levels of the majority of genes are stable across samples (arrays), rescaling by assuming various profiles have the same median (or trimmed mean) intensity measures works quite well. However, when we have less than 2,000 miRNAs being tested and the majority of miRNAs are weakly or not expressed, the performance of such a normalization method could be quite questionable.

Efforts have been devoted to finding specific controls for miRNA normalization. In ideal situations, controls should be consistently stable and highly abundant despite tissue types or treatments for a specific analytical platform. Also, they should have characteristics similar to miRNAs, including size, biogenesis and stability. Non-coding RNAs (ncRNAs) have been utilized as normalization controls by some arrays, including Exiqon miRCURY LNA miRNA Array, Luminex FlexMIR panel and TaqMan-based qRT-PCR, as well. However, it is found that some ncRNA normalization controls can be influenced by chemo drug treatments, such as 5-FU, Cisplatin or Doxorubicin [37,39,40]. As a result, we need to be aware of the stability of normalization controls across a relatively wide variety of tissues, cell lines and conditions. When a number of normalization controls are available to use, it is recommended to evaluate their stability validation before they are adopted for data normalization. For the FlexmiR bead arrays, we proposed a measurement error model-based algorithm to normalize the intensity measures from the five different pools by using the four normalization beads [41].

Efforts have also been made to normalize miRNA microarray data using “invariants”—a set of miRNAs that are not differentially expressed across arrays [38,42,43,44,45,46]. Because the actual expression levels cannot be determined in different molecular units, it is also challenging to determine whether a set of miRNAs are actually not changing or stably changed across arrays. In other words, if there are two groups of miRNAs having similar performances, it might be tricky to decide which group can be used for normalization. Wang *et al*. proposed to borrow the strength of another platform and estimate the overall expression pattern of the entire miRNA profile using a panel of representative miRNAs validated with qRT-PCR results [47].

### 4.2. Nonlinear Normalization

It has been observed that the changes of gene expression levels are nonlinear, especially for those highly expressed genes/miRNAs. Loess normalization is a popular normalization method for miRNA microarrays, which is based on robust local regression of the log ratios of the intensity measures from two arrays (or two differently-dyed samples from the same array) on the overall spot intensities. Variants of the loess normalization method has also been introduced so as to refine the linear scaling part to enhance its performances [48,49,50]. Quantile normalization is another commonly used nonlinear normalization method, which has been successfully migrated to miRNA array data analysis. The quantile normalization method is proposed under an assumption that there is an underlying common distribution of intensities of all miRNAs across arrays [51,52,53]. For miRNA arrays, due to the small total number of miRNAs and the overall low expression level, the ranks of the miRNAs could be greatly affected by the background noises, and hence, the performances of the quantile normalization method could be affected. However, quantile normalization is reported in the literature as one of the best performed normalization methods for miRNA data [37,40,41,45,46,47]. For the FlexmiR bead arrays, due to the small number of miRNAs profiled in the five pools (60, 64,64, 65 and 66), the quantile normalization is not suitable for intra-sample (among pools) normalization. Though, after the sub-profiles have been appropriately assembled, the quantile normalization method has relatively better performance than some other normalization methods [41].

## 5. Differentially-Expressed miRNA Detection

In miRNA microarray data analysis, the main interests are usually focused on whether a specific miRNA or a set of miRNAs are differentially expressed in the two cell populations. The concept of “differentially-expressed” is not well-defined, which makes it challenging to detect the differentially expressed miRNAs. Without loss of generality, we assume an miRNA has expression, *x*, in the control sample, and *y*, in the test sample. Usually, we will judge whether the miRNA is differentially expressed by checking the fold-change (FC), which is the ratio of y/x. If FC=1, the miRNA is not differentially expressed. If FC is much larger than 1.0, we say the miRNA is upregulated; otherwise, if FC is much smaller than 1.0, we say the miRNA is downregulated. An miRNA that is either downregulated or upregulated is said to be differentially expressed. However, it is not clear how different that can be treated as differentially expressed. We need to take both the fold-change and the basal level of the miRNA into consideration. For example, if the miRNA is highly expressed, a two-fold change might be practically meaningful, and the miRNA can be considered as differentially expressed. Meanwhile, if the miRNA is weakly expressed, a large FC value might be practically meaningless to the researchers. In practice, we do not know the actual value of FC; instead, we estimate it using $\hat{FC}=g_{1}\left(X\right)/g_{2}\left(Y\right)$. Here, $g_{1}\left(.\right)$ and $g_{2}\left(.\right)$ are two functions that are expected to be able to calibrate the measurement errors and will approximate the true values of *x* and *y*, respectively. As shown in the measurement quality and background correction section, the majority of miRNAs have low SNRs, and thus, the measurement errors could play very important roles in the intensity measures, *Y* and *X* and, hence, could severely influence the estimate of the fold-change. In other words, measurement errors pose a higher degree of uncertainty in identifying the differentially expressed miRNAs. We need to use the FC with caution in differentially-expressed miRNA detection. The fold-change criteria should be used only to miRNAs that are turned on (“expressed”) in both samples. In more detail, if an miRNA is expressed in one sample, but not expressed in the other sample, the FC value could be zero or infinity, in theory. In practice, due to the existence of background signal, the observed FC value could be very small or very large. In such cases, it is relatively easy to detect the differentially-expressed miRNAs, though such miRNAs should be dealt with separately, because they will make the distribution of FC values of all miRNAs very skewed. While in another scenario, if an miRNA is not expressed in both samples, the FC value is dominated by background noise, and such miRNAs should be excluded from the study.

In miRNA discovery studies, when a large number of replicates are available, statistical tests, such as *t*-test, ANOVA or other omnibus tests can be used for differentially expressed miRNA detection. It is worth noting that the non-linear normalization methods, including the loess method and quantile normalization method, are preferred, due to their capability of dealing with the nonlinear changes of miRNAs with different expression levels. Fan *et al.* proposed a model with a parametric component and a non-parametric component to test the differentially expressed genes by taking the treatment effect, block (position) effect and the nonlinear relationship into consideration [54].

In practice, many laboratories do not repeat array experiments. As a result, there is no biological replicates for each miRNA. For some microarray platforms, there might be a few technical replicates available on each array. The lack of sufficient replicates makes it a big challenge to identify the differentially expressed miRNAs. For instance, in the 48 LNAv9 arrays, the ten osterosarcoma xenograft specimens are treated with three chemo-drugs and saline, respectively. Most of the samples are tested once, and some are repeated two times. If research interests are focused on the drug-resistance of different patients to the three chemo drugs, respectively, there is only one or no biological replicate. For the LNAv7 and LNAv9 arrays, there are four technical replicates on each array for every miRNA. However, for the bead arrays, there is neither a biological replicate nor technical replicates. In [40], the authors proposed to identify the differentially expressed miRNAs by regressing the expressions from the test sample on the expressions from the control sample using an errors-in-variables non-parametric regression model. This method can be applied to detect the differentially expressed miRNAs based on two arrays without replicates. When replicated samples or probes are available, data from various arrays can be integrated and, hence, improve the overall performance of the regression model to detect differentially expressed miRNAs, which is also validated using the qRT-PCR results [40].

The measurement error model-based tests can make good use of the global information from each of the expression profiles, and the required number of samples per gene/miRNA can be greatly reduced [18,19]. However, when the overall signal quality of an array is fair or poor, the outcomes could be quite questionable if no biological replicate is available to use. It is crucial to have multiple biological replicates (at least two) to improve the sensitivity and specificity in differentially-expressed miRNA detection.

## 6. Materials and Methods

### 6.1. HCT-116 Cell Lines

Two cell lines of HCT-116 (wt-p53 and null-p53) were treated with three drugs: 5-fluorouracil (5-FU), oxaliplatin (OX) and irinotecan (CPT-11). The total RNAs were isolated from the six treated cell lines and two non-treated cell lines (serving as controls) and tested with miRCURY LNA MicroRNA Array v7.5.0 (Exiqon Inc.). For HCT-116 (null-p53), the control sample was tested three times, and the CPT-11 treated sample was tested twice. For HCT-116 (wt-p53), both the control sample and the 5-FU treated sample were tested twice. A total of 359 miRNAs were profiled for each of the 13 cell lines. A total of 37 miRNAs were randomly selected and further tested using TaqMan-based qRT-PCR on an ABI 7500HT instrument (Applied Biosystems Inc., Foster City, CA, USA) [9,47,55,56,57].

### 6.2. Osterosarcoma Xenograft Specimens

Ten human osterosarcoma xenograft specimens were collected, and each was treated with saline (as control) and three chemotherapeutic treatments: cisplatin (CIS), doxorubicin (DOX) and ifosfamide (IFO). The total RNAs were isolated from each sample and analyzed with the following three platforms: (a) miRCURY LNA MicroRNA Array (Exiqon Inc.; Vedbaek, Denmark, based on miRbase 9.2); (b) Luminex FlexmiR MicroRNA Human Panel; and (c) TaqMan Array Human MicroRNA Panel (Applied Biosystems,Foster City, CA, USA, v2.0). A total of 577 human miRNAs were profiled with the LNA array, 391 with the Luminex bead array and 664 with the TaqMan array, where a total of 213 miRNAs were shared by all three platforms [37,40,41,58,59].

### 6.3. Generalized Logarithm Transformation

The following two-component measurement error model is proposed to model the measured expression levels,

(1) $$y=\alpha +\mu e^{\eta }+\epsilon$$

where *y* is the measured raw expression level, *α* is the mean background noise, *μ* is the true expression level and *η* and *ϵ* are the multiplicative and additive measurement errors, which are assumed to be normally-distributed with mean 0 and variances $\sigma_{\eta }^{2}$ and $\sigma_{\epsilon }^{2}$, respectively [17,18,19]. The variance of *y* under this model is $Var\left(y\right)=\mu^{2}S_{\eta }^{2}+\sigma_{\epsilon }^{2},$ where $S_{\eta }^{2}=e^{\sigma_{\eta }^{2}}\left(e^{\sigma_{\eta }^{2}}-1\right)$. To ease the analyses of gene-expression microarrays using some standard statistical techniques, the following generalized logarithm transformation that stabilizes the variance has been proposed:

(2) $$f_{c}\left(z\right)=\ln\left[\frac{z+\sqrt{z^{2}+c^{2}}}{2}\right]$$

where $c=\sigma_{\epsilon }/S_{\eta }$. The performance of the GLOG is further studied, and simulation results show that it is a better choice compared with the “started logarithm” transformation and the “log-linear hybrid” transformation [20,60,61,62,63,64,65,66].

## 7. Conclusions

As a well-established discovery tool in biological and biomedical research, microarray has been successfully migrated to miRNA studies. Different microarray platforms have their own strengths and weaknesses. In ideal situations, we can make good uses of various microarray platforms to borrow the strengths from each other. However, the reality is that most researchers tried to stay with as few platforms as possible, due to the low reproducibility among arrays across platforms and/or across laboratories. The diversity of miRNA microarray platforms and lack of reliable analytical methods actually have made cross-platform miRNA microarray data comparison and integration challenging.

Measurement errors exist in all microarray platforms. In miRNA microarrays, the expressions of numerous miRNAs might be dominated by measurement errors, and the overall signal quality of the miRNA microarrays is usually deficient. Thus, it is especially important that the miRNA microarray experiments will be well designed and well conducted. In some literature, it was emphasized to ensure the measurement quality via controlling the experimental factors, and it was suggested that the background signal subtracted signals should be used directly without any normalization for miRNA microarray data [1,67,68]. However, we need to be aware that measurement errors are inevitable in microarray experiments. Conclusions based on microarray data without proper normalization might be quite misleading.

Before we apply any statistical method to normalize microarray data for further analysis or to calibrate measurement errors to detect differentially expressed miRNAs, it is always good to screen first the bad microarrays off—if evidence shows that an array completely failed, it should be excluded from further analyses. To our knowledge, there is no existing method to evaluate systematically the quality of different slides. However, we can assess the quality of various arrays via the following different ways. First, if the experiment is conducted well, a reasonably large portion of strong signals are expected from each array. One of the LNAv9 array contains only 3.89% of spots that are not flagged. This is a strong indication that the whole slide might not be usable. Automatic flagging can be applied to check the signal quality. However, we should not simply discard the measures from those probes that are flagged as weak. On the other hand, if a probe is found to be contaminated, the corresponding measure should not be used. Second, we can compute the Spearman’s correlation coefficient between any pair of arrays. The Spearman’s correlation coefficient is rank based, so it won’t be affected by the distributions of the miRNA expressions in the same array. For example, arrays tested with the same platform are supposed to have reasonably high reproducibility. A very small Spearman’s correlation coefficient usually can be used as an indicator of something wrong in the array data. Third, we can compare the density distributions of expressions of all miRNAs being tested in every array. For miRNA microarrays, due to the small total number of miRNA and also the violation of the assumption that the numbers of upregulated and downregulated miRNAs are approximately the same, it is hard to judge the signal quality of an array when it has a different expression distribution.

Normalization is an essential matter for microarray data analysis. A well-developed normalization method can efficiently calibrate the measurement errors and can offer a powerful tool for cross-platform and cross-laboratory microarray data integration. Most existing normalization methods for miRNA microarray data are adopted from mRNA/cDNA microarrays with or without modifications. However, the unique signature of miRNA has reduced the enthusiasm of such adoption. In our previous studies and research by others, it has been found that the two-component measurement error model and the generalized logarithm transformation work well for microarray data. Based on the measurement error model, several normalization methods and differentially-expressed miRNA detection algorithms have been developed and achieved good results. We should keep in mind that the measurement errors coupled with data from different platforms have different characteristics, and hence, it is not realistic to develop one or a few methods that can deal with data from all platforms. For example, expression data obtained from bead arrays have stronger signals, but without technical replicates. As a result, we have to heavily count on the global information from the profile for bead array when there is no replicated array for the same specimen. In addition, most of the existing normalization methods based on the global information are not applicable to normalize the sub-profiles, due to the extreme small number of miRNAs tested in each pool. When dealing with the traditional glass-based arrays, which have weaker signals compared with the bead arrays, but usually have several technical replicates, special attention needs to be paid to the background correction and how to find robust estimates from several replicates on each array. It is also worth noting that borrowing the strengths of some reliable analytical platforms, such as the TaqMan Array Human MicroRNA Panel, might be a good approach for miRNA microarray data normalization. Meanwhile, we also need to keep an eye on the quality of qRT-PCR results as well—measurement errors also exist in qRT-PCR platforms. The qRT-PCR results should be used as “gold standards” with caution.

We recommend to keep the measurements from the weakly expressed miRNAs in the analysis. However, in detecting the differentially expressed miRNAs, miRNAs expressed at different levels should be tested (viewed) differently. According to their expressions in the control sample, the miRNAs can be grouped into three groups: group 1, for those with expression significantly stronger than the background noise; group 2, for those with expression close to the background noise; and group 3, for all others. For miRNAs in group 3, it is meaningless to test whether any of them are downregulated, and attention should be paid more to those in the other two groups, especially in group 1.

In summary, it is challenging, but necessary, to develop some novel adaptive statistical methods to efficiently calibrate the measurement errors for normalization and for differentially-expressed miRNA detection. In that way, we can reuse the miRNA microarray data saved in a variety of databases and to integrate data from similar studies contributed by different laboratories using various platforms. Even in an era in which the next-generation deep sequencing technologies have been widely used, microarray is still very valuable as a reliable and affordable profiling tool. In addition, measurement errors and bias exist in small RNA sequencing data, too. Some of the normalization methods for miRNA, including the quantile normalization, smoothing-based normalization methods and measurement error model-based normalization methods, can also be applied to small RNA sequencing data with or without modifications.
